# Estimating Client Out-of-Pocket Costs for Accessing Voluntary Medical Male Circumcision in South Africa

**DOI:** 10.1371/journal.pone.0164147

**Published:** 2016-10-26

**Authors:** Michel Tchuenche, Vibhuti Haté, Dacia McPherson, Eurica Palmer, Ananthy Thambinayagam, Dayanund Loykissoonlal, Emmanuel Njeuhmeli, Steven Forsythe

**Affiliations:** 1 Health Policy Project, Project SOAR (Supporting Operational AIDS Research), Avenir Health, Washington, DC, United States of America; 2 Project SOAR (Supporting Operational AIDS Research), George Washington University, Washington, DC, United States of America; 3 Health Policy Project, Palladium Consultant, Johannesburg, South Africa; 4 USAID (United States Agency for International Development), Pretoria, South Africa; 5 National Department of Health, Pretoria, South Africa; 6 USAID (United States Agency for International Development), Washington, DC, United States of America; Cardiff University, UNITED KINGDOM

## Abstract

In 2010, South Africa launched a countrywide effort to scale up its voluntary medical male circumcision (VMMC) program on the basis of compelling evidence that circumcision reduces men’s risk of acquiring HIV through heterosexual intercourse. Even though VMMC is free there, clients can incur indirect out-of-pocket costs (for example transportation cost or foregone income). Because these costs can be barriers to increasing the uptake of VMMC services, we assessed them from a client perspective, to inform VMMC demand creation policies. Costs (calculated using a bottom-up approach) and demographic data were systematically collected through 190 interviews conducted in 2015 with VMMC clients or (for minors) their caregivers at 25 VMMC facilities supported by the government and the President’s Emergency Plan for AIDS Relief in eight of South Africa’s nine provinces. The average age of VMMC clients was 22 years and nearly 92% were under 35 years of age. The largest reported out-of-pocket expenditure was transportation, at an average of US$9.20 (R 100). Only eight clients (4%) reported lost days of work. Indirect expenditures were childcare costs (one client) and miscellaneous items such as food or medicine (20 clients). Given competing household expense priorities, spending US$9.20 (R100) per person on transportation to access VMMC services could be a significant burden on clients and households, and a barrier to South Africa’s efforts to create demand for VMMC. Thus, we recommend a more focused analysis of clients’ transportation costs to access VMMC services.

## Introduction

South Africa has the highest number of people living with HIV in the world. As of 2015, about 7 million people—nearly 12.96% of South Africa’s population—were HIV-positive [1]. Consequently, HIV prevention and treatment has long been the foremost priority for the National Department of Health (NDOH). Based on strong evidence that voluntary medical male circumcision (VMMC) reduces men’s risk of acquiring HIV through heterosexual intercourse by 60% [2], South Africa began scaling up its national VMMC program in 2010 [2–4]. Medical circumcision procedure takes about 20–30 minutes. However, WHO guidelines recommend that recently circumcised men abstain from sexual activity for 6 weeks to ensure complete healing [5].

Slightly more than 45% of males in South Africa over the age of 15 were circumcised as of 2014 [6, 7]. According to an article in this collection, using updated HIV incidence projections based on new HIV surveillance data and assuming achievement of the 90-90-90 treatment goals of the Joint United Nations Programme on HIV/AIDS (UNAIDS), South Africa will avert 94,000 new HIV infections by 2025 if 80% VMMC coverage was reached by 2015 and is maintained through 2025 [8]. The 2020 UNAIDS goals are 90 percent of all those with HIV to have been diagnosed, 90 percent of those diagnosed to be on antiretroviral treatment (ART), and 90 percent of those on ART to be virally suppressed [9]. This ambitious 80% VMMC coverage target translates to 4.3 million circumcisions to be performed by the end of 2016 in South Africa [10, 11, 12].

The same article in this collection also notes that from 2010 through 2014, about 9 million VMMCs had been conducted in 14 priority countries, representing 43% of the estimated 20.9 million VMMCs required to reach 80% coverage by the end of 2015 [8]. VMMC demand creation—mobilizing and motivating men to accept and access VMMC services—is crucial to reach this ambitious target. In 2014, VMMC demand creation expenditures ($14.2 million) [13] represented about 26% of all resources that the President’s Emergency Plan for AIDS Relief (PEPFAR) spent on VMMC in South Africa [14]. Given this large investment, why is uptake low? What might hinder clients’ access to the service?

Providing good-quality VMMC services is essential to increasing the national coverage of circumcisions. Another important factor is accelerating the uptake of services specifically within the South Africa program’s target population: males ages 15–49. VMMC is offered free of charge at public-sector health facilities in South Africa; however, most clients seeking VMMC services are incurring indirect out-of-pocket expenses such as transportation cost or foregone income. Research has shown that a household’s out-of-pocket expenditures for healthcare can hinder its long-term income-generating capabilities [15]. Because these costs could discourage uptake of VMMC services within the target population in South Africa, we conducted a detailed investigation of clients’ out-of-pocket expenses, in order to inform the country’s demand creation policies.

The objective of this study, therefore, is to provide useful information to the National Department of Health and VMMC stakeholders regarding the scope of financial barriers that VMMC clients might face. Information on the cost to clients also provides insights into economic barriers that boys (and their caregivers) and men can encounter when they seek VMMC services. This is the first study to estimate out-of-pocket and opportunity costs to clients seeking VMMC services in South Africa.

The most common VMMC modes of service delivery in South Africa are fixed (static) sites, fixed sites with outreach services, and mobile services. By understanding the financial and economic barriers to VMMC service uptake, policy and health decision-makers may deploy these services in ways more likely to overcome these barriers and therefore increase uptake of services.

## Methods

### Ethical Considerations

The Human Research Ethics Committee (Medical) of the University of the Witwatersrand, South Africa, and the Health Media Lab Institutional Review Board, in Washington DC—which is authorized by the U.S. Department of Health and Human Services (DHHS), Office of Human Research Protections, and which has DHHS Federal-Wide Assurance approval—provided ethical approvals for this costing study.

### Client Surveys

Client cost data were systematically collected and analyzed from 25 Government of South Africa- and PEPFAR-supported VMMC facilities (fixed sites and fixed sites with outreach services) in eight of South Africa’s nine provinces (see S1 Table). Mobile sites intended to bring VMMC services close to clients either were not operational during the study period or were not accessible without additional levels of approval.

To assess the types of costs incurred by clients and their families (e.g., expenses incurred or income lost by the VMMC client or his caregiver), semi-structured interviews were conducted with qualitative and quantitative capture components (the survey instrument is provided in S1 Appendix). Study participants were selected using purposive sampling. VMMC clients were identified based on specific demographic and service utilization characteristics that allowed for the most relevant and accurate information to be gathered. These characteristics are:

1. Only clients coming in for a follow-up visit post-surgery were interviewed. Clients who were at the facility for pre-surgery or surgery visits were not interviewed, because the aim of this study was to collect all costs related to accessing VMMC services: the costs of pre-surgery visits, surgery visits, and post-surgery visits.
2. Clients were selected from the sample of returning clients at the facility on a given day willing to be interviewed. The study protocol determined that at least three clients had to be present at the facility for selection by facility staff (that is, data collectors did not select any of the respondents).
3. The client to be interviewed was not next in line for an appointment and clients or caregivers had at least 30 minutes or more before their appointments to ensure that there was sufficient time to complete the questionnaire.

Researchers administered the informed consent form to prospective respondents, allowing them to accept or decline the interview. If the client was under 16 and was accompanied by a caregiver, the latter was interviewed instead of the minor client. Overall, there was a 100% response rate; none of the selected clients or their caregivers refused to participate.

The surveys were conducted from April–May, 2015, which falls within South Africa’s season of low VMMC uptake (August–May). The facilities were selected based on an initial random sampling of three sites in each province. However, the list of selected sites was subsequently modified (reduced, increased, or replaced partially or entirely with new sites) by provincial authorities, who noted that some sites were not fully functional (e.g., they had only recently begun operation, were not regularly seeing VMMC clients, or had completely stopped VMMC operations). This is why there is a discrepancy in the number of sites per province, and some provinces have fewer than three sites (see S1 Table).

Opportunity costs of travel and post-operative healing times to the client and/or his caregiver were assessed through days of missed work (because recovery after the medical procedure can take up to seven days). This information was calculated using the clients’ monthly income bracket and disaggregated by clients’ age, province, facility type, and facility location, and by service modality. Qualitative and quantitative data from the client surveys were transcribed as discrete responses and notes per coded interview.

Data collection aimed to answer a central question: What (out-of-pocket and opportunity) costs are incurred by clients seeking VMMC services in South Africa? The data collection tool was developed exclusively for the purpose of this study and pre-tested at a facility in Gauteng (see S1 Appendix). The surveys had three sections: (1) direct medical expenses, (2) direct nonmedical expenses, and (3) indirect expenses. In the direct medical expenses section, background demographic data of clients and their insurance and billing information were documented. The direct nonmedical expenses section reviewed the arrangements for a client’s clinic visits, transportation costs for these visits, and other associated costs, such as food and wound-care products. The section on costs not directly related to accessing VMMC services covered lost income from employment and other sources and the costs for childcare, home care, or other tasks that had to be undertaken as a result of required modifications in the schedule of the client or caregiver as a result of the VMMC procedure.

## Results

### Client Background Information

**Fig 1:**
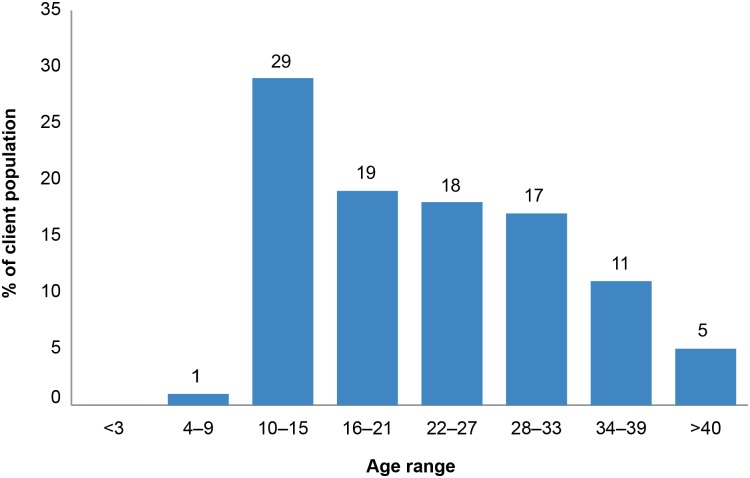
Distribution of VMMC clients interviewed by age.

Of the 190 interviews conducted, the average age of all clients interviewed was 22 years. The average caregiver age was 42 years. Most of the respondents were the clients themselves; 166 respondents (87.4%) were clients while 24 (12.6%) were caregivers. Fig 1 provides a detailed representation of the distribution of the surveyed VMMC clients’ ages.

The data showed that about half of VMMC clients interviewed who were circumcised at the surveyed government- and PEPFAR-supported sites in 2014 were under the age of 22. While nearly 92% of clients interviewed were under 35 years of age, only 29% of total clients were between 10 and 15 years of age. Of note, it was reported that some sites erroneously turn away potential VMMC clients who were 10–14 years old, even though South Africa’s policy is not to deny the service to anyone. This implies that reported percentages for this age group could be significantly higher if demand from this age group were fully absorbed.

While most clients were unaccompanied, caregivers accompanied minor clients. About 46% of caregivers (11 out of the reported 24 caregivers) were over 49 years of age; younger caregivers, such as those 20–24 years old, generally were older siblings.

### Expenses Incurred by Clients and Caregivers

The main costs reported by clients and for minor clients by their caregivers were transportation costs. While other specific costs related to accessing male circumcision services were reported, such as the cost of meals and materials for wound treatment, in several instances data labeled “other expenses” had no further explanation and thus were insufficiently detailed for inclusion in this analysis. Of course, because the service is free to clients in South Africa’s public health facilities, the cost of the circumcision itself was reported as nil.

#### Transportation Costs

**Fig 2:**
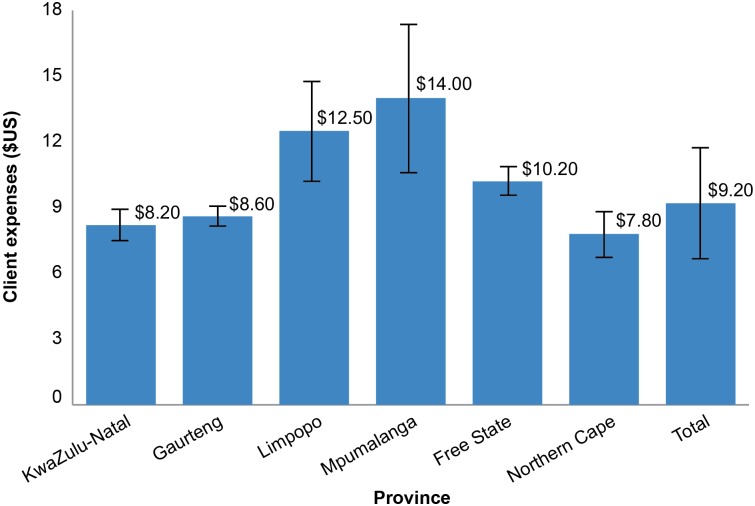
Average transportation expenses by Province.

The average round-trip transportation expense to receive VMMC services (from pre-surgery to estimated follow-up visits when applicable) for all respondents reporting transportation costs was US$9.20 (R100). All dollar amounts are U.S. currency. Of the clients and caregivers (reporting for their minor clients) interviewed, 54.20% (103/190) reported that they incurred travel expenses; 45.80% did not respond. For those who did not respond, there was no way to determine if the respondent did not know the answer to the question or if they incurred no costs for transportation. The average cost of transportation presented excludes those who did not provide a response. There was considerable variation in transportation costs by province, ranging from an average of $7.75 (R84) in Northern Cape to approximately $14.00 (R152) in Mpumalanga (Fig 2).

**Fig 3:**
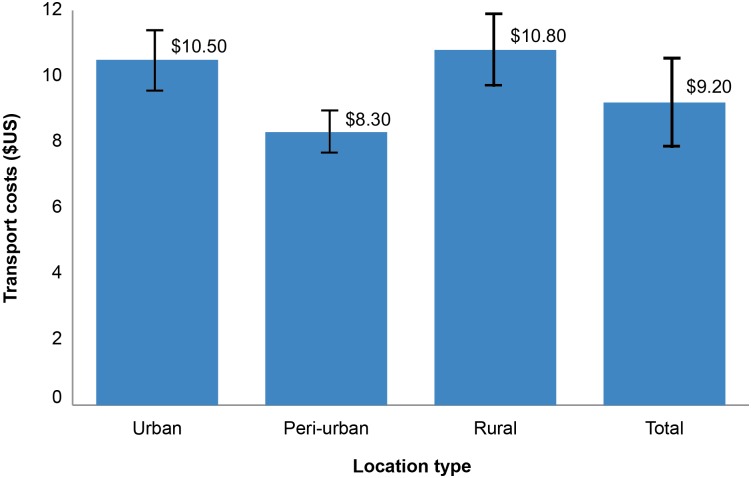
Transportation costs by location.

Transportation costs incurred by VMMC clients were almost identical at urban and rural facilities, with mean M = $9.88 (see Fig 3). This cost was approximately $10.53 (R114) in urban areas and $10.80 (R114) in rural areas. Clients accessing services at peri-urban facilities spent about $8.31 (R90) for transportation, which is slightly less than what their urban and rural counterparts pay for transportation to access VMMC services.

#### Lost Wages

In addition to transportation costs, respondents (clients and caregivers of minor clients) reported lost days of work and other expenses, including the costs of meals and (for clients only, of course) wound care. Respondents were asked to report the number of days of work missed due to VMMC and if they had lost any income due to those missed days of work. The data showed that 21% (40) reported missing days of work but only 4% (8) reported wages lost.

#### Other Reported Expenses

Other sources of indirect expenditure were childcare and miscellaneous items such as food or medicine. However, only one client reported requiring childcare to access VMMC services and 20 clients (11%) reported expenditure on food and medicines. Furthermore, the information on food and medicine varied greatly, such that it was difficult to undertake rigorous analysis. It should be noted that the main source of other expenses was salt for wound care (applied topically in solution with water to cure the wound), at an average price of $2 (R21).

#### Study limitations

Recall bias is one of the main limitations of this study. As clients were at various stages of follow-up, the time from when they incurred the expenses they reported also varied, possibly influencing reporting accuracy.

Clients and caregivers were neither asked who paid for their transportation nor to disaggregate their reported transportation costs (caregiver vs minor client). Because some PEPFAR partners do pay clients’ transportation, this study could not capture how the 44.8% of clients who did not report incurring any cost for transportation got to the facilities. It is therefore impossible to know:

- Who covered their transportation cost?
- Were they living near the health facility, and thus did not require transportation?
- Were they the beneficiaries of VMMC outreach services?

Only 3 clients reported being covered by any (public or private) health insurance. Thus, any costs incurred by VMMC clients with insurance coverage were not statistically viable. Also, the survey did not collect annual VMMC insurance costs and/or VMMC bills received from health insurance providers or interview clients at private facilities.

The limited sample size could introduce some bias, leading to underrepresentation of certain groups of clients. High use of services is in winter: June–July. Therefore, some differences in out-of-pocket expenditures may exist in other months.

Finally, information is lacking on clients who wanted circumcision but could not afford the associated costs. Our survey data only provided information on VMMC clients who sought circumcision at public facilities. Useful follow-up studies would be a household survey of potential clients who are not currently accessing VMMC services.

## Discussion

Surveyed clients seeking VMMC services who incurred transportation costs reported that they had on average spent US9.20 (R100). This figure covers pre-surgery visits through all follow-up visits. With respect to lost income, respondents (clients and minors’ caregivers) were asked to report the number of days of work missed due to VMMC and if they lost any income due to those lost days of work. The data showed that 21% (40) reported missing days of work but only 4% (8) reported wages lost. These findings mean that:

- A majority of VMMC adult clients and minors’ caregivers are unemployed, which is not surprising given the relatively young age of the VMMC clients.
- VMMC adult clients and minor clients’ caregivers who are employed probably have benefits such as sick leave or other leave that allow them to seek services without losing wages.

While lost income did not appear to represent a significant burden either for adult clients or caregivers, it is worth noting that interviews were conducted only with those who have determined that the burden of pursuing circumcision was not significant enough to prevent them from seeking services. It is possible, for example, that older males might be discouraged from seeking services due to a potential loss of income. Our findings do not speak to such a barrier, because the research focused only on adults who had already sought and received the VMMC procedure and the caregivers of minors who had done so.

## Conclusion

The largest VMMC client out-of-pocket expenditure is transportation costs, despite considerable variability among provinces. Though circumcision itself is free, clients and caregivers both incur significant transportation costs. In 2011, poor households in South Africa spent about $2.12 (R23) a day—a third of their daily income—on food and $0.70 (R7) a day on transportation. The share of daily income of non-poor households for these expenses was 10%: $3.50 (R38) on food and $5.80 (R63) on transportation [16]. Given competing household expenditure priorities that are difficult to forgo (e.g., housing and utilities expenditures, which represent about 20% of a poor household’s annual expenditures), spending $9.20 (R100) per person on transportation to access VMMC services could represent a significant barrier to the expansion of demand for VMMC. We recommend a more focused analysis of transportation costs. For instance, because costs were slightly lower at peri-urban facilities, it would be worthwhile to determine:

- Whether most clients who receive care at peri-urban facilities generally live closer to or have cheaper transportation to these facilities than do clients who receive care at urban or rural facilities
- If VMMC uptake by those in a facility's catchment area is higher for facilities that subsidize transportation than for facilities that do not

This study is the first to estimate out-of-pocket expenses incurred by clients accessing the free VMMC service at public-sector health facilities in South Africa. It provides VMMC demand creation and communication policymakers key information on a potential barrier that could hinder the uptake of VMMC services. Other countries with ambitious VMMC targets could find the results of this study beneficial, because achieving milestone VMMC goals will require understanding potential barriers to VMMC service uptake.

## Supporting Information

S1 Appendix VMMC Clients Data Collection Instrument. (DOCX)

S1 Table VMMC Clients Survey Sites. (DOCX)
