# Supplementary material for: Estimating Client Out-of-Pocket Costs for Accessing Voluntary Medical Male Circumcision in South Africa
Source: PLoS One. 2016 Oct 26;11(10):e0164147. doi: 10.1371/journal.pone.0164147 (PMC5082609; doi:10.1371/journal.pone.0164147)
Supplement: S1 Appendix — (DOCX) [file pone.0164147.s001.docx]

**Addendum S1. VMMC Clients Data Collection Instrument**

**CLIENT COST DATA COLLECTION FORM**

This is a 2-part survey with multiple question subsets. The survey contains 11 pages. Some pages and sections may be skipped, as guided by the survey, according to client answers.

The survey is estimated to take anywhere from 20 to 55 min to implement, depending on the interviewee’s responses. On average, it is hoped the survey will not take much more than 30 minutes and this is communicated to the interviewees in the consent form.

Time estimates appear below with section descriptions.

**PART 1A: DIRECT MEDICAL EXPENSES ASSESSMENT (4–14 min)**

1. ***Interview Questions Set 1: Basic background about the person you are interviewing, the client and the current visit.* (2 min)**
2. ***Interview Questions Set 2: Insurance and bills.* (2–12 min)**

**Part 1B: DIRECT NON-MEDICAL EXPENSES ASSESSMENT (7–23 min)**

1. ***Interview Questions Set 1: Background on facility visits and associated transportation costs.* (5–15 min)**
2. ***Interview Questions Set 2: Associated costs other than transportation.* (2–8 min)**

**Part 2: INDIRECT EXPENSES AND OPPORTUNITY COST ASSESSMENT (10–25 min)**

1. ***Interview Questions Set 1: Employment and missed income of the person you are interviewing (either the client if he is 18+ or the caregiver).* (5–8 min)**

***SUPPLEMENT: FILL THIS SECTION ONLY IF CLIENT IS A MINOR AND YOU ARE SPEAKING TO HIS CAREGIVER* (3–7 min)**

***Interview Questions Set 1 Supplement: Missed income and*** opportunity of the client, if the client is a minor (<18 years).

1. ***Interview Questions Set 2: Employment and missed income of others related to the person you are interviewing.* (5–10 min)**

***Don’t forget to record the facility, time, date and interviewer information on the paper forms before you begin the interviews.***

**Facility: ____________________ Interviewer: ___________________ Time: _________________ Date: ________________**

***Instructions and Informed Consent***

*Please administer the informed consent form to the client (or the caregiver if the client is under 18) and obtain their signature/mark. Confirm you have only captured follow-up clients.*

*After consent is obtained, in the following forms please mark X where applicable and leave notes in space provided as relevant.*

**PART 1A: DIRECT MEDICAL EXPENSES ASSESSMENT**

***Interview Questions Set 1: Basic background about the person you are interviewing, the client and the current visit.***

1. Are you the client that received the male circumcision procedure?

 YES.

 NO. If NO, please describe your relation to the client:______________________________

1. How old is the client?______________________________

How old is the caregiver (if applicable)?______________________________

1. What is today’s visit for? __________________________________________________________
2. When did the client have the circumcision procedure done (day/month/year, where possible)?

__________(day) __________(month) __________(year)

***Interview Questions Set 2: Insurance and bills.***

1. Is the client currently covered by health insurance?

 NO. If NO, please describe how you normally pay for your hospital or doctors’ visits:

 Out-of-pocket, and/or

 Other (describe):______________________________________________________

 YES. If YES, how much do you pay for your health insurance?

______________monthly or ______________yearly.

If YES, what is the name of the insurance provider?_____________________________

If YES, how long have you had this provider? _________________________________

If YES, why did you choose this particular provider? ____________________________

If YES, did male circumcision influence at all your decision to either choose this provider or remain with this insurance provider (describe if so)?

________________________________________________________________

1. Did you receive any bill(s) for male circumcision, including pre-surgery visits, the day of surgery and/or at any follow-up appointments?

 NO.

 YES. If YES, please list the full bill amount(s) below to the best of your ability.

Please record the materials and services per bill either from the bill itself or as per the client’s confirmation. If materials/services appear itemized with amounts

on a bill, please note the itemized costs along with their descriptions. If the client does not have any bills to present, ask them to recall to the best of their ability how many bills they have received and the approximate amounts and dates of those bills. If they recall what the bills were for, that can be added in the notes.

| Bill 1 Amount | Bill 1 Date |
| --- | --- |
| Bill 1 Description of Materials and Services | |

| Bill 2 Amount | Bill 2 Date |
| --- | --- |
| Bill 2 Description of Materials and Services | |

| Bill 3 Amount | Bill 3 Date |
| --- | --- |
| Bill 3 Description of Materials and Services | |

| Bill 4 Amount | Bill 4 Date |
| --- | --- |
| Bill 4 Description of Materials and Services | |

**Part 1B: DIRECT NON-MEDICAL EXPENSES ASSESSMENT**

***Interview Questions Set 1: Background on facility visits and associated transportation costs.***

1. How many total visits to a facility of any type have you (and/or the client) had regarding male circumcision, including pre-surgery, testing and/or counseling, the surgery itself, post-surgery follow-up, etc?

________________________________________________________________

1. Is this facility the same facility where you/the client had your/their male circumcision procedure?

 YES.

 NO. If NO, where did you/the client get the VMMC procedure done? _________________________________________________________________________

Why are you visiting this facility instead of where you received the procedure?

________________________________________________________________

1. Please use the boxes below to name all the facilities you have been to regarding VMMC, including pre-surgery preparations, operation visit, follow-up visits and/or complication visits. Please note the reason you went to each facility listed, how much you paid to get there and if you/the client went alone or brought someone with you/them.

PRE-SURGERY VISITS

| **Visit 1** | |
| --- | --- |
| Facility Name | Visit Date |
| Visit Reason | |
| Round-Trip Transportation Cost *(clarify that each round-trip cost this amount, e.g. a caregiver and client together would have to pay this 2X)* | |
| Who did the traveling *(describe relationship to client, if not only the client himself)* | |

| **Visit 2** | |
| --- | --- |
| Facility Name | Visit Date |
| Visit Reason | |
| Round-Trip Transportation Cost *(clarify that each round-trip cost this amount, e.g. a caregiver and client together would have to pay this 2X)* | |
| Who did the traveling *(describe relationship to client, if not only the client himself)* | |

| **Visit 3** | |
| --- | --- |
| Facility Name | Visit Date |
| Visit Reason | |
| Round-Trip Transportation Cost *(clarify that each round-trip cost this amount, e.g. a caregiver and client together would have to pay this 2X)* | |
| Who did the traveling *(describe relationship to client, if not only the client himself)* | |

SURGERY VISIT

| Facility Name | Visit Date |
| --- | --- |
| Visit Reason | |
| Round-Trip Transportation Cost *(clarify that each round-trip cost this amount, e.g. a caregiver and client together would have to pay this 2X)* | |
| Who did the traveling *(describe relationship to client, if not only the client himself)* | |

COMPLETED POST-SURGERY VISITS *(include today’s visit and all other visits completed so far)*

| **Visit 1** | |
| --- | --- |
| Facility Name | Visit Date |
| Visit Reason | |
| Round-Trip Transportation Cost *(clarify that each round-trip cost this amount, e.g. a caregiver and client together would have to pay this 2X)* | |
| Who did the traveling *(describe relationship to client, if not only the client himself)* | |

| **Visit 2** | |
| --- | --- |
| Facility Name | Visit Date |
| Visit Reason | |
| Round-Trip Transportation Cost *(clarify that each round-trip cost this amount, e.g. a caregiver and client together would have to pay this 2X)* | |
| Who did the traveling *(describe relationship to client, if not only the client himself)* | |

| **Visit 3** | |
| --- | --- |
| Facility Name | Visit Date |
| Visit Reason | |
| Round-Trip Transportation Cost *(clarify that each round-trip cost this amount, e.g. a caregiver and client together would have to pay this 2X)* | |
| Who did the traveling *(describe relationship to client, if not only the client himself)* | |

1. Please summarize the total number of visits for VMMC there has been:

__________Pre-Surgery Visits

__________Surgery Visit *(this should be 1. Visits for any complications in which the procedure had to be performed again should be recorded as a Post-Surgery Visit).*

__________Completed Post-Surgery Visits

__________Known Future Post-Surgery Visits

If there are any discrepancies between the number reported here and those captured in the above boxes for Question 3 and the figure reported in Question 1 of this question set please describe why:

_________________________________________________________________________

_________________________________________________________________________

***Interview Questions Set 2: Associated costs other than transportation.***

1. Aside from transportation costs, have you incurred any of the following additional costs related to traveling to a health facility for male circumcision, including before, during and after surgery? (please check the box where applicable and describe in space provided)

 Arranging child-care support while you are away from the home?

- If so, how much did this cost in total so far?__________________________________

- And if you have future appointments how much do you expect you will have to pay for child-care beyond what you have already for child care?________________________

_____________________________________________________________________

 Arranging other support while you are away from home or work?

- If so, please describe what this was and how much did this cost in total so far?

______________________________________________________________________

- And if you have future appointments how much do you expect you will have to pay for this support beyond what you have already?__________________________________

_____________________________________________________________________

 Cost of lodging or extra meals for you/your party if you had to travel away from the

home and stay overnight to receive VMMC services?

- If so, please describe what this was and how much did this cost in total so far?

______________________________________________________________________

- And if you have future appointments how much do you expect you will have to pay

for lodging or extra meals beyond what you have already?_______________________

_____________________________________________________________________

1. Please use this space to describe the amounts and details of any other costs associated with getting male circumcision done that you don’t think we covered:

|  |
| --- |

**Part 2: INDIRECT EXPENSES AND OPPORTUNITY COST ASSESSMENT**

This section of the survey is to better help us understand costs other than direct medical and transportation expenses, such as missed opportunities and missed wages that you/the client may have incurred in choosing to undergo male circumcision.

***Interview Questions Set 1: Employment and missed income of the person you are interviewing (either the client if he is 18+ or the caregiver).***

1. Which range best describes your monthly total income, from all forms of employment and/or public assistance?

 R0-R900

 R900-R1,300

 R1,300-R2,300

 R2,300-R4,200

 R4,200-R9,800

 R9,800-R18,000

 R18,000-R25,700

 R25,700+

1. Are you employed?

 YES.

 NO.

If NO, do you receive any income in the form of public assistance, cash from friends,

etc? Please describe type and amount: ______________________________________

______________________________________________________________________

1. If you are employed, how many days of work have you missed so far due to VMMC?___________

3a. If you missed any days of work, will you lose any income due to those missed days of

work?

 NO.

 YES. If YES, how much?_______________________________________________

1. Will you miss any more days of work in the future due to VMMC until you are/the client is completely recovered?

 NO.

 YES/PROBABLY. If YES/PROBABLY, how many?__________________________

 NOT SURE.

4a. If you will miss any days of work in the future, will you lose any income due to those missed days of work?

 NO.

 YES/PROBABLY. If YES/PROBABLY, how much?___________________________

 NOT SURE.

1. Whether you are employed or unemployed, please describe any additional hardship or missed opportunity you feel you experienced due to the VMMC procedure or appointments? _________________________________________________________

| ***SUPPLEMENT: FILL THIS SECTION ONLY IF CLIENT IS A MINOR AND YOU ARE SPEAKING TO HIS CAREGIVER***  ***Interview Questions Set 1 Supplement: Missed income and opportunity of the client, if the client is a minor (<18 years old).***   1. Does the client minor earn any income?    NO.   YES.  If YES, describe how this income is earned: __________________________________  If YES, describe how much he earns per month: _______________________________  1a. To date, has the client minor forgone any of this income due to his circumcision planning or appointments?   NO.   YES. If YES, how much?______________________________________________   1. Will the client minor lose any more income in the future due to circumcision appointments, etc?    NO.   YES. If YES, how much?___________________________________________________   NOT SURE.   1. Whether the client minor earns any income or not, please describe any additional hardship or missed opportunity he/you feel he experienced due to the VMMC procedure or appointments?   _____________________________________________________________________ |
| --- |

***Interview Questions Set 2: Employment and missed income of others related to the person you are interviewing.***

1. Has anyone else missed days of work so far due to any of your VMMC-related appointments?

 NO. *(skip to question 2 if NO)*

 YES. If YES, how many people __________ and how are they each related to you?

______________________________________________________________________

______________________________________________________________________

1a. Also if YES, how many days of work did each of the people you list miss?

______________________________________________________________________

______________________________________________________________________

1b. And did the person/people you listed lose any income due to missed days of work?

 NO.

 YES. If YES, how much (please specify by each person listed)?

________________________________________________________________

________________________________________________________________

1c. Also, if YES, please specify the income bracket that best describes each person you mentioned (mark multiple times and note names if applicable):

 R0-R900__________________________________________________

 R900-R1,300______________________________________________

 R1,300-R2,300_____________________________________________

 R2,300-R4,200_____________________________________________

 R4,200-R9,800_____________________________________________

 R9,800-R18,000____________________________________________

 R18,000-R25,700___________________________________________

 R25,700+_________________________________________________

1d. Is this person/are any of these people unemployed?

 NO.

 YES. If YES, do they receive any income in the form of public assistance,

cash from friends, etc? Please describe type and amount for each person:

(space provided next page)

________________________________________________________________

1. Please specify how many days of work, if any, each person listed above will miss in the future until you are/the client is completely recovered

_________________________________________________________________________

2a. If others will miss any days of work in the future, will they lose any income due to those

missed days of work?

 NO.

 YES. If YES, how much (please specify by each person listed)?

______________________________________________________________________

1. Finally, please describe here if you or anyone else lost any other form of income/earnings or experienced any particular hardship not yet captured in this survey due to you/the client’s preparation for MC, undergoing MC surgery or going to follow-up appointments, etc:

|  |
| --- |

**END. Thank you for your participation!**
