# Supplementary material for: Estimating Client Out-of-Pocket Costs for Accessing Voluntary Medical Male Circumcision in South Africa
Source: PLoS One. 2016 Oct 26;11(10):e0164147. doi: 10.1371/journal.pone.0164147 (PMC5082609; doi:10.1371/journal.pone.0164147)
Supplement: S1 Table — (DOCX) [file pone.0164147.s002.docx]

**Table S1. VMMC Clients Survey Sites**

| Gauteng | Jubilee District Hospital |
| --- | --- |
|  | Kgabo Clinic |
|  | Laudium Community Health Centre |
|  | ODI District Hospital |
|  | Oliven Clinic |
|  | Phedisong Clinic |
|  | Suurman Clinic |
| KwaZulu-Natal | Kwadabeka Community Health Centre |
|  | Itshelejuba District Hospital |
|  | Stanger District Hospital |
|  | Kwamashu Polyclinic |
|  | Newtown |
|  | Benedictine District Hospital |
|  | Northdale District Hospital |
|  | Mpumelelo Clinic |
| Limpopo | Mapela Clinic |
|  | Mogoto Primary Health Care Clinic |
|  | Evelyn Lekganyane Primary Health Care Clinic |
| Mpumalanga | Embhuleni Hospital |
|  | Witbank Regional Hospital |
|  | Mapulaneng Regional Hospital |
| North West | Letlhabile Community Health Clinic |
| Free State | Lesedi Community Health Centre |
| Northern Cape | Galeshewe Day Hospital |
| Western Cape | Malmesbury |
